# Early percutaneous tracheotomy in coronavirus disease 2019 (COVID-19) and infection in healthcare personnel: a cohort study

**DOI:** 10.1017/ice.2020.1399

**Published:** 2021-01-05

**Authors:** Antonio Rosano, Enrico Martinelli, Federica Fusina, Alessandro Morandi, Michele Bertelli, Elena Malpetti, Pierluigi Ferretti, Carmine R. Militano, Marco Marri, Giuseppe Natalini

**Affiliations:** 1 Department of Anesthesia and Intensive Care, Fondazione Poliambulanza Istituto Ospedaliero, Brescia, Italy; 2 Department of Information and Communications Technology, Fondazione Poliambulanza Istituto Ospedaliero, Brescia, Italy

*To the Editor—*Early tracheotomy is associated with shorter intensive care unit (ICU) stay compared to late tracheotomy,^1^ and this procedure could therefore be useful in a context of severely limited resources like the one observed during the novel coronavirus disease 2019 (COVID-19) pandemic.^2^ Nevertheless, tracheotomy in COVID-19 patients is considered risky for healthcare workers. In absence of evidence, guidelines and recommendations advise avoiding or delaying tracheotomy in COVID-19 patients.^3–5^

In this study, we assessed whether early percutaneous tracheotomy was associated with an increased risk of severe acute respiratory syndrome coronavirus-2 (SARS-CoV-2) infection for healthcare staff. Data were collected in patients admitted to the ICU of Fondazione Poliambulanza hospital in Brescia (Italy) from February 20, 2020, to May 5, 2020. Two cohorts of healthcare workers were identified: (1) the exposed cohort included doctors and nurses who participated in the early percutaneous tracheostomy procedure as first operator, fiberoscopist, instrumental or anesthesia nurse and (2) the nonexposed cohort included staff on duty in the COVID-19 ICU who never participated in the procedure.

Infection of staff by SARS-CoV-2 was identified using a positive reverse-transcriptase real-time polymerase chain reaction (PCR) test from a nasopharyngeal swab or in presence of IgM or IgG for SARS-CoV-2 in the serum (antibody test). The observation period after the last tracheotomy was 30 days. A nasopharyngeal swab was mandatory if body temperature (measured before each work shift) was >37.5°C and in staff complaining of symptoms compatible with COVID-19 or who had been absent due to illness. Moreover, all healthcare personnel were invited to undergo blood testing for SARS-CoV-2 (both Abbott SARS-CoV-2 IgG chemiluminescent microparticle immunoassay, Abbot Laboratories, Chicago, IL, and Coretest COVID-19 IgM/IgG Ab Test, Core Technology, China) as a surveillance measure. Staff infections were considered to be related to the exposure if the timing of the infection was subsequent to the first exposure. Data on SARS-CoV-2–infected staff were anonymized.

Patients were evaluated for percutaneous tracheotomy after the first 3 days of mechanical ventilation if weaning from mechanical ventilation could not be reasonably completed within the following 7 days.^6^ All tracheotomies were performed at the bedside with a percutaneous single-dilator technique and were guided by fiberoptic bronchoscopy. The involved personnel comprised 2 doctors (first operator and fiberoscopist) and 2 nurses (the instrumental nurse and the anesthesia nurse who assisted with airway management for fiberoscopy). The first operator was always a senior doctor, well experienced in percutaneous tracheostomy. The operator and instrumental nurse were equipped with a sterile surgical gown over the disposable protective gown, surgical gloves on disposable protective gloves, filtering face piece 3 (FFP3) respirator, surgical mask, visor, and cap. The doctor performing the fibroscopy and the anesthesia nurse were protected by a disposable protective gown, double nonsterile gloves, FFP3 respirator, surgical mask, visor, and cap. Ventilation was never paused during the procedure.

The study outcome was to compare the rate of infection with SARS-CoV-2 between the cohort of staff exposed and the cohort not exposed to the tracheotomy procedures.

Data are shown as mean (standard deviation), median (IQR, first–third quartile), or frequency (percentage). Frequencies were compared using the Fisher exact test. Data management and statistical analyses were performed using R version 3.6.1 software (R Foundation for Statistical Computing, Vienna, Austria).

The protocol was approved by Brescia’s ethics committee.

We performed 121 early percutaneous tracheotomies on the 181 patients admitted to the ICU with COVID-19. Most patients were male (n = 93, 77%), and the median age was 64 years (SD, 9). Hospital mortality was 45.5%. Tracheotomy was performed on median day 6 (IQR, 5–7) of ICU stay.

In total, 145 ICU staff members (58 doctors and 85 nurses) participated in the care of COVID-19 patients, and 91 of these (63.6%) were in the exposed cohort. Overall, 132 staff members (92%) underwent serological testing to detect SARS-CoV-2 IgM/IgG.

In total, 15 healthcare workers (11.4%) were infected with SARS-CoV-2, without a significant difference between doctors and nurses (9.3% vs 12.8%; *P* = .59). Table 1 summarizes the comparison between the rates of SARS-CoV-2 infection in workers exposed and not exposed to tracheotomy procedures. Exposed staff did not have an increased rate of infection compared to nonexposed staff, neither when considered as an entire group nor when the analysis was stratified by the role that staff members played in the tracheotomy procedures. In the same study period, 37 of 37 doctors (100%) in the exposed cohort also performed tracheal intubation in COVID-19 patients in the ICU or operating room, compared with 17 of 21 of doctors (81%) in the cohort not exposed to tracheostomy (*P* = .01)

**Table 1.** Comparison Between the Rate of SARS-CoV-2 Infection in Workers Involved and Not Involved in Tracheotomy Procedures

Characteristic	Frequency/Total (%)	*P* Value^a^
Exposed to tracheotomy with any role Not exposed to tracheotomy procedure	7/91 (7.7) 6/52 (11.5)	.55
**Subgroups**		
Exposed to tracheotomy as first operator Not exposed to tracheotomy as first operator	0/6 (0) 13/137 (9.5)	1
Exposed to tracheotomy as fiberoscopist Not exposed to tracheotomy as fiberoscopist	0/35 (0) 13/108 (12)	.04
Exposed to tracheotomy as instrumental nurse Not exposed to tracheotomy as instrumental nurse	4/24 (16.7) 9/119 (7.6)	.23
Exposed to tracheotomy as anesthesia nurse Not exposed to tracheotomy as anesthesia nurse	5/44 (11.4) 8/99 (8.1)	.54

a *P* ≤ .05 was considered statistically significant.

Our findings indicate that early percutaneous tracheotomy did not expose healthcare personnel to an increased risk of SARS-CoV-2 infection. Doctors in the cohort of those exposed to tracheotomy had a higher frequency of involvement in tracheal intubation procedures in COVID-19 patients, but the infection rate for this cohort did not increase.

Tracheotomy is defined as early if it is performed within 10 days of tracheal intubation.^1^ Percutaneous tracheotomies were performed within the first 10 days in 98% of our patients, while the latest procedure was performed after 12 days. Early percutaneous tracheotomy can offer an organizational advantage compared to the surgical one because procedures can be performed at the bedside.^8^ This can be particularly useful in conditions of high demand, when almost all of the operating rooms are being used as ICU stations.

In conclusion, early percutaneous tracheotomy, even when performed in COVID-19 patients, appears to be safe for healthcare workers when personal protective equipment is used.
